# Three new bamboo-feeding species of the genus *Symplanella* Fennah (Hemiptera, Fulgoromorpha, Caliscelidae) from China

**DOI:** 10.3897/zookeys.408.5797

**Published:** 2014-05-10

**Authors:** Lin Yang, Xiang-Sheng Chen

**Affiliations:** 1 Institute of Entomology & Special Key Laboratory for Development and Utilization of Insect Resources, Guizhou University, Guiyang, Guizhou Province 550025, P. R. China; 2 College of Animal Sciences, Guizhou University, Guiyang, Guizhou Province 550025, P. R. China

**Keywords:** Fulgoroidea, bamboo planthopper, morphology, Oriental region, taxonomy

## Abstract

Three new species of the Oriental caliscelid planthopper genus *Symplanella* Fennah, *S. hainanensis* **sp. n.**, *S. recurvata* **sp. n.** and *S. zhongtua* **sp. n.**, are described and illustrated from South China. A checklist and a key to species of genus *Symplanella* are provided.

## Introduction

The genus *Symplanella* was erected by Fennah (1987) based on specimens from Burma (type species: *Symplanella breviceps* Fennah, 1987) and was placed in the subtribe Augilina of the tribe Ommatidiotini of the family Issidae. Recently, the genus was transferred to the family Caliscelidae by Gnezdilov and Wilson (2006) when they reviewed the family Caliscelidae. Zhang and Wang (2009) reviewed the species of *Symplanella* from China and described one new species, *Symplanella unipuncta* Zhang & Wang, 2009, and proposed one new combination, *Symplanella brevicephala* (Chou, Yuan & Wang, 1994) (transferred from *Symplana* Kirby). To date, only three species, *Symplanella brevicephala* (China: Yunnan), *Symplanella breviceps* (Burma: Dawna Hills) and *Symplanella unipuncta* (China: Hainan), are included in the genus *Symplanella*.

In this paper three new species of the genus *Symplanella* are described and illustrated from South China (Guangdong, Guangxi, Hainan and Yunnan). The generic characteristics are redefined. A checklist and a key to known species of *Symplanella* are provided.

## Materials and Methods

Terminology follows Fennah (1987) and Chan and Yang (1994). Dry specimens were used for the descriptions and illustrations. External morphology was observed under a stereoscopic microscope and characters were measured with an ocular micrometer. Measurements are given in millimeters; body length is measured from the apex of the head to the apex of the forewing in repose. The genital segments of the examined specimens were macerated in 10% KOH, washed in water and transferred to glycerine. Illustrations of the specimens were made with a Leica MZ 12.5 stereomicroscope. Photographs were taken with a Leica D-lux 3 digital camera. The digital images were then imported into Adobe Photoshop 8.0 for labeling and plate composition. The type specimens and material examined are deposited in the Institute of Entomology, Guizhou University, Guiyang, China (IEGU).

## Taxonomy

### Symplanella

Fennah, 1987

http://species-id.net/wiki/Symplanella

Figs 1 – 36

Symplanella Fennah, 1987: 244; Zhang and Wang 2009: 176.

#### Type species.

*Symplanella breviceps* Fennah, 1987, by original designation.

#### Diagnosis.

Vertex (Figs 3, 15, 27) with anterior margin angular or rounded, posterior margin angulately concave, disc distinctly depressed, without median carina. Frons (Figs 5, 17, 29) with median carina and submedian carinae, longer in middle line than widest part, widest at level of second segment of antennae. Clypeus with median carina. Pronotum as broad as or broader than head including eyes; lateral carinae strongly diverging laterad. Mesonotum without carina, almost twice as broad as long. Forewing (Fig. 6) long and narrow, 4.00–4.71 times as long as broad; Sc+R and M united in basal fifth, Sc+R forking close to nodal transverse line; three or four subapical cells and seven to nine apical cells; M with three or four branches. Hindwing broad triangular, venation as shown in Fig. 7. Post-tibia with one spine laterally, six apically; basal and second metatarsal segments toothless and ventrally pilose. Abdomen exceptionally elongate, narrow sternites chevron-shaped. Genital styles narrow and long or short and oval. Aedeagus fused with connective, both forming V-shaped or Y-shaped; aedeagal shaft long, simple, phallobase slender lobe-like or reduced.

**Figures 1–12.**
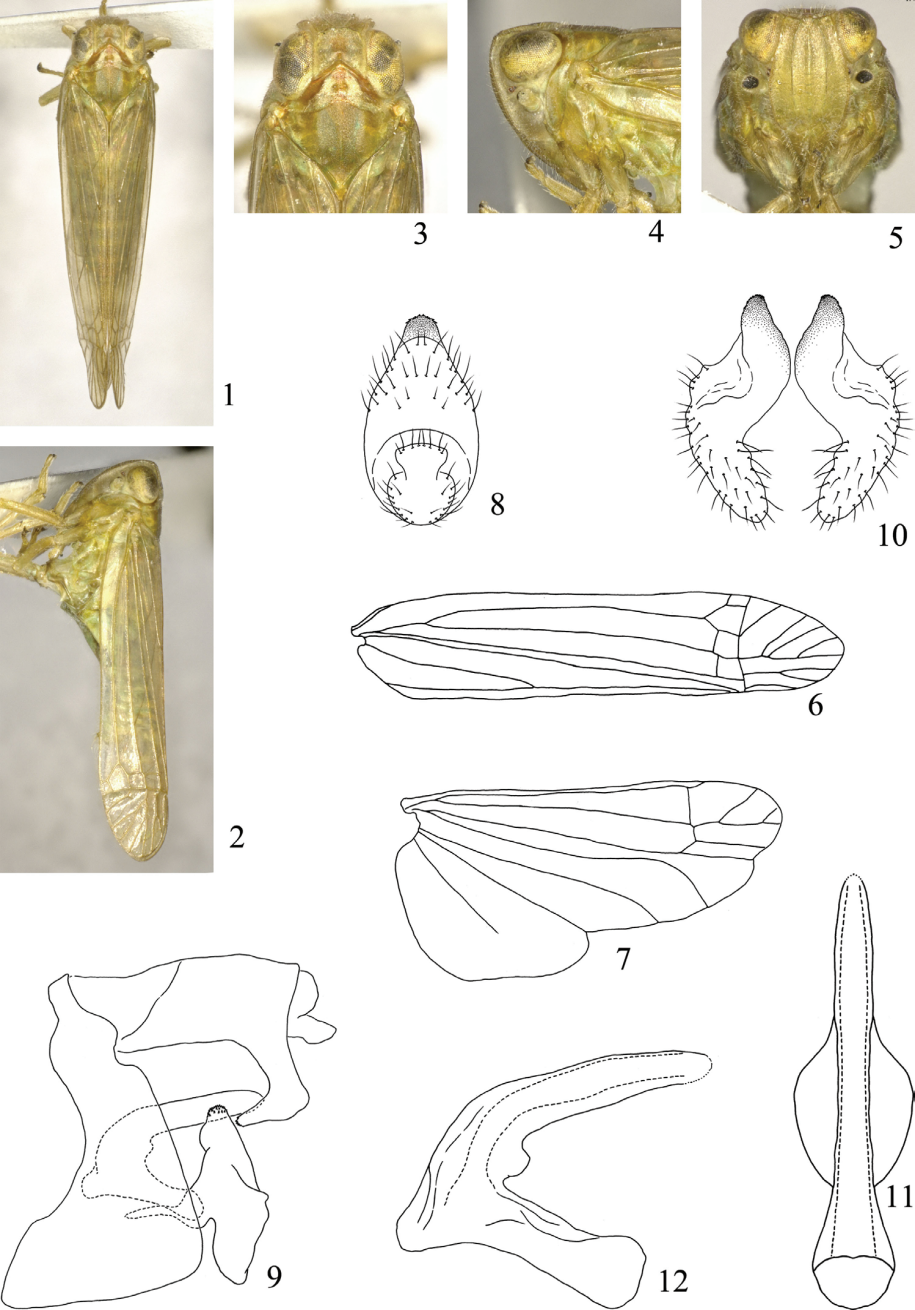
*Symplanella recurvata* sp. n. **1** Male habitus, dorsal view **2** Male habitus, lateral view **3** Head and thorax, dorsal view **4** Head and thorax, lateral view **5** Face **6** Forewing **7** Hindwing **8** Male anal segment, posterior view **9** Male genitalia, lateral view **10** Styles, posterior view **11** Aedeagus, dorsal view **12** Aedeagus, lateral view.

**Figures 13–24.**
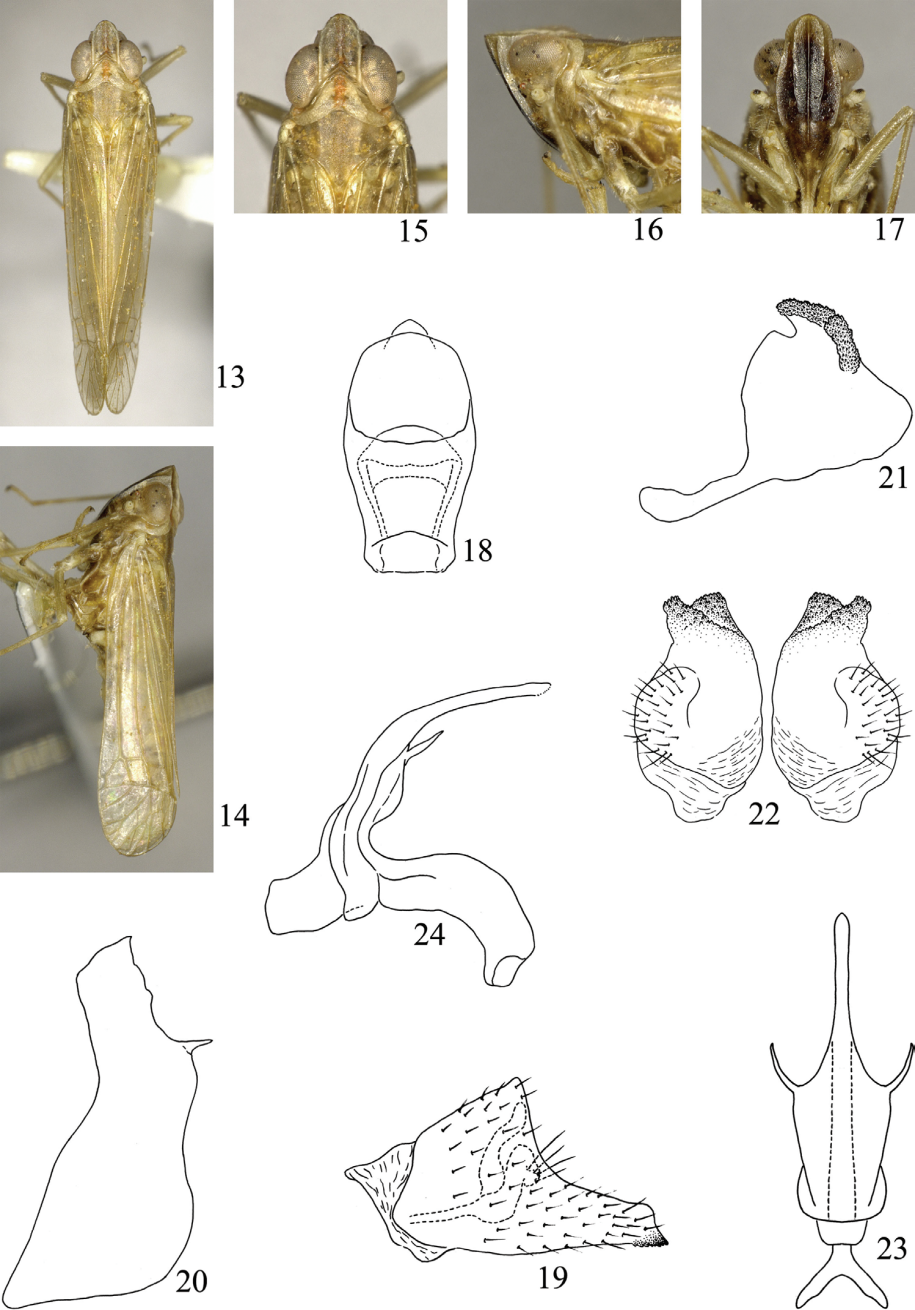
*Symplanella hainanensis* sp. n. **13** Male habitus, dorsal view **14** Male habitus, lateral view **15** Head and thorax, dorsal view **16** Head and thorax, lateral view **17** Face **18** Male anal segment, dorsal view **19** Male anal segment, lateral view **20** Male pygofer, lateral view **21** Style, lateral view **22** Styles, posterior view **23** Aedeagus, dorsal view **24** Aedeagus, lateral view.

**Figures 25–36.**
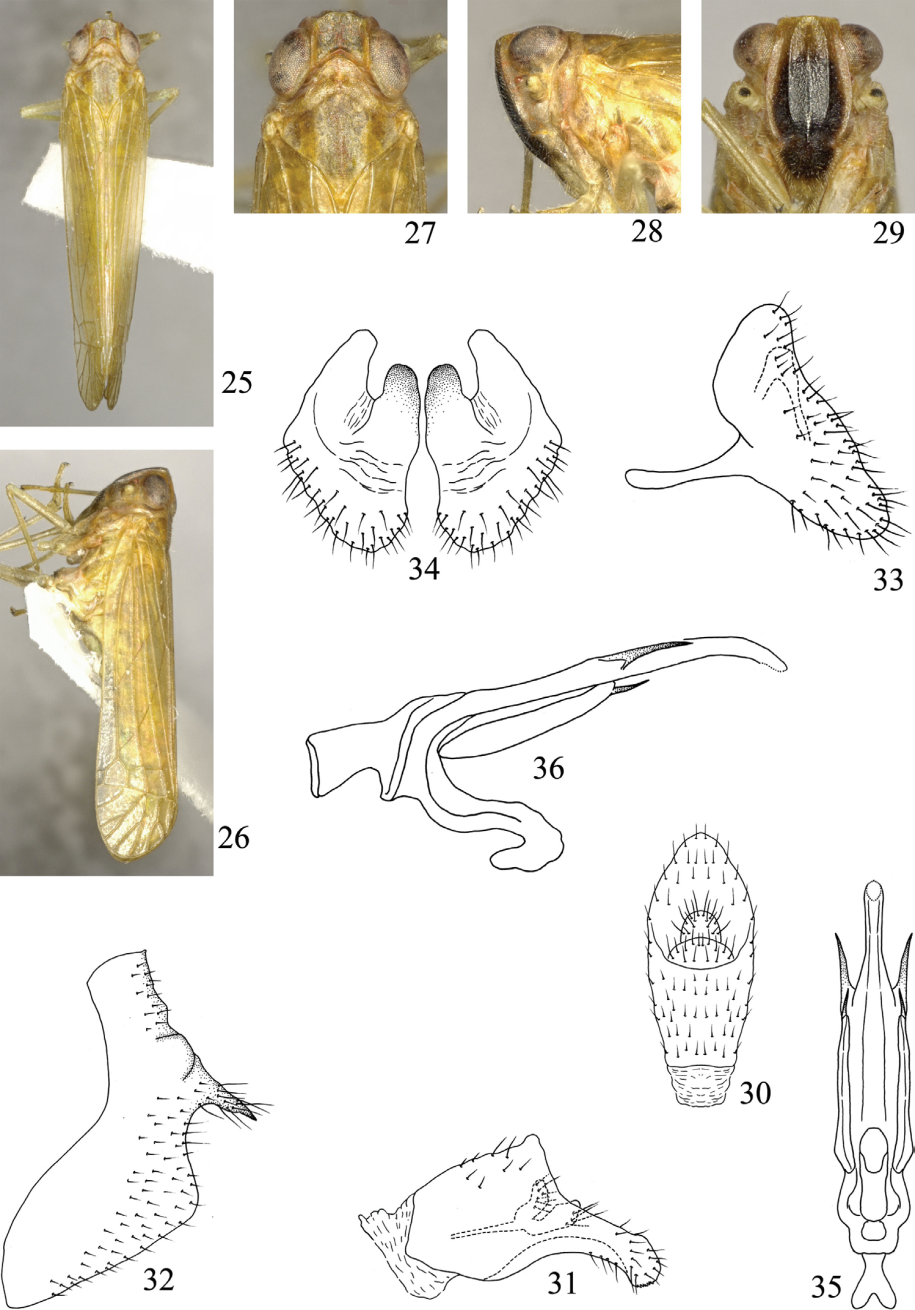
*Symplanella zhongtua* sp. n. **25** Male habitus, dorsal view **26** Male habitus, lateral view **27** Head and thorax, dorsal view **28** Head and thorax, lateral view **29** Face **30** Male anal segment, dorsal view **31** Male anal segment, lateral view **32** Male pygofer, lateral view **33** Style, lateral view **34** Styles, posterior view **35** Aedeagus, ventral view **36** Aedeagus, lateral view.

#### Distribution.

Oriental Region (China and Burma).

#### Checklist of species of *Symplanella* Fennah, 1987

*Symplanella brevicephala* (Chou, Yuan & Wang, 1994); China (Yunnan).

*Symplanella breviceps* Fennah, 1987; Burma (Dawna Hills).

*Symplanella hainanensis* sp. n.; China (Hainan).

*Symplanella recurvata* sp. n.; China (Guangdong and Guangxi).

*Symplanella unipuncta* Zhang & Wang, 2009; China (Hainan).

*Symplanella zhongtua* sp. n.; China (Yunnan)

#### Key to species of genus *Symplanella* (male)

**Table:** 

1	Frons and clypeus mostly blackish or dark brown (Figs 17, 29)	2
–	Frons and clypeus mostly yellowish green (Fig. 5)	4
2	Head in lateral view with the apex acute (Fig. 16)	*Symplanella hainanensis* sp. n.
–	Head in lateral view with the apex rounded (Fig. 28)	3
3	Frons and clypeus mostly blackish brown (Fig. 29); pygofer with one stout process at middle (Fig. 32)	*Symplanella zhongtua* sp. n.
–	Frons and clypeus mostly dark brown; pygofer with one lobe-like process at dorsal posterior angle	*Symplanella brevicephala*
4	M of forewing with three branches; genital style long in posterior view, posterior margin broadly concave	*Symplanella breviceps*
–	M of forewing with four branches (Fig. 6); genital style short in posterior view (Fig. 10), posterior margin not concave or slightly convex (Fig. 9)	5
5	Posterior margin of pygofer with one process	*Symplanella unipuncta*
–	Posterior margin of pygofer without process (Fig. 9)	*Symplanella recurvata* sp. n.

### Symplanella recurvata sp. n.

http://zoobank.org/FA3249B6-4106-4928-9201-F444D3E76BED

http://species-id.net/wiki/Symplanella_recurvata

Figs 1–12

#### Measurements.

Body length including forewing: male 5.78–5.98 mm (N = 6), female 6.15–6.25 mm (N = 12); forewing length: male 4.90–5.15 mm (N = 6), female 5.30–5.40 mm (N = 12).

#### Coloration.

General color light yellowish brown with somewhat green. Ocelli reddish brown, eyes black brown. Antennae with one black spot at apex of second segment. Central area of vertex and pronotum, base of mesonotum with somewhat pale yellowish red. Procoxae, mesocoxae, metapleura, abdominal sternites laterally and pregenital sternite of female fuscous.

#### Head and thorax.

Vertex including eyes narrower than pronotum (0.86:1). Vertex shorter in middle line than broad at base (0.60:1). Frons 1.28 times longer in middle line than widest part. Pronotum slightly longer in middle line than vertex (1.21:1). Mesonotum 1.24 times as long as vertex and pronotum together in middle line. Forewing longer in middle line than broad at widest part (4.71:1). Hindwing longer in middle line than broad at widest part (2.01:1), venation as shown in Fig. 7.

#### Abdomen.

Anal segment of male in posterior view (Fig. 8) nearly long oval, in lateral view (Fig. 9) with basal half parallel dorsally and ventrally, apical margin produced into stout process ventrally, which curves cephalad, apex acutely rounded. Pygofer in lateral view (Fig. 9) with dorsal margin distinctly shorter than ventral margin, posterior margin mostly straight, concave at dorso-posterior angle. Genital style in lateral view slender, dorsal and ventral angles acutely rounded, posterior margin slightly convex; in posterior view (Fig. 10) with dorsal and ventral apex acute and rounded, inner and outer margin sinuate. Aedeagus in dorsal view (Fig. 11) with shaft straight, simple, apex rounded; in lateral view (Fig. 12) curved at basal third, apical part straight, phallobase small, lobe-like. Connective in lateral view (Fig. 12) straight, fused with base of aedeagus forming V-shape; connective in dorsal view (Fig. 11) with both lateral margins swelled laterad.

#### Type material.

Holotype: ♂, China: Guangdong, Guangzhou, Huanan Botanical Garden (23°08'N, 113°14'E), on bamboo (*Neosinocalamus* sp.), 22 Nov. 2006, X.-S. Chen; paratypes: 5 ♂♂, 11 ♀♀, data same as holotype; 1 ♀, Guangxi, Daxin, Encheng, 4 May 2009, H.-R. Li.

#### Host plant.

Bamboo (*Neosinocalamus* sp.).

#### Distribution.

**Figure 37.**
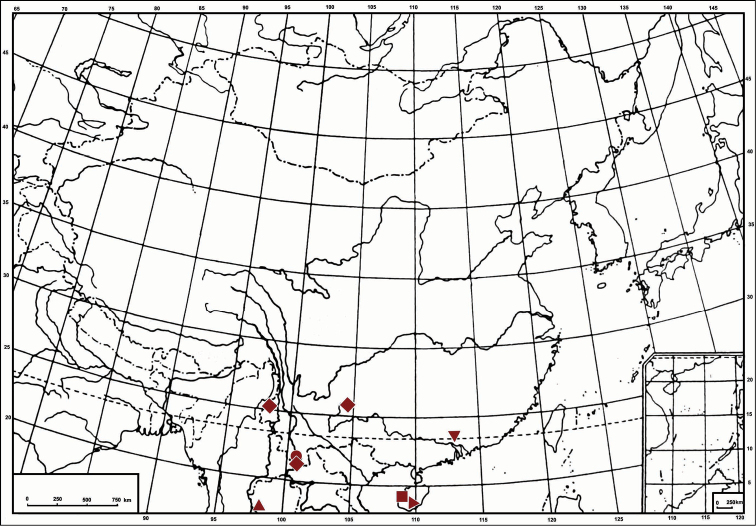
Geographic distribution of *Symplanella* species: *Symplanella recurvata* sp. n. (▼); *Symplanella brevicephala* (Chou, Yuan & Wang) (♦); *Symplanella breviceps* Fennah (▲); *Symplanella hainanensis* sp. n. (►); *Symplanella unipuncta* Zhang & Wang (■); *Symplanella zhongtua* sp. n. (●).

South China (Guangdong and Guangxi) (Fig. 37).

#### Remarks.

This new species is closely related to *Symplanella unipuncta* Zhang & Wang, 2009 but differs in: *i*) anal segment in lateral view with one stout process at apical margin ventrally, which curves cephalad apically (with one tooth-like process at middle of ventral margin in *Symplanella unipuncta*); *ii*) posterior margin of pygofer without process (with one process in *Symplanella unipuncta*); *iii*) aedeagus without process at base (with two long stout processes basally and ventrally, which as long as aedeagal shaft).

#### Etymology.

The new species is named after the strongly recurved tip of the anal tube.

### Symplanella hainanensis sp. n.

http://zoobank.org/C12B4D8F-BF4B-4939-977B-D13F7CEDDF9F

http://species-id.net/wiki/Symplanella_hainanensis

Figs 13–24

#### Measurements.

Body length including forewing: male 5.45–5.62 mm (N = 2), female 6.00–6.30 mm (N = 8); forewing length: male 4.40 mm (N = 2), female 4.65–4.95 mm (N = 8).

#### Coloration.

General color dirty yellowish brown. Ocelli reddish brown, eyes black brown. Antennae with one black spot at apex of second segment. Frons and clypeus mostly dark brown. Central area of vertex and pronotum, base of mesonotum with somewhat pale yellowish red. Procoxae, mesocoxae, metapleura, abdominal sternites laterally and pregenital sternite of female, fuscous.

#### Head and thorax.

Vertex including eyes as wide as pronotum. Vertex longer in middle line than broad at base (1.24:1). Frons 1.67 times longer in middle line than widest part. Pronotum shorter in middle line than vertex (0.74:1). Mesonotum 0.87 times as long as vertex and pronotum together in middle line. Forewing longer in middle line than broad at widest part (4.00:1).

#### Abdomen.

Anal segment of male in dorsal view (Fig. 18) with median part slightly widened; in lateral view (Fig. 19) broad at basal half, abruptly narrowed at apical half, apex acute, with some micro teeth at apical ventral margin. Pygofer in lateral view (Fig. 20) with dorsal margin distinctly shorter than ventral margin, posterior margin sinuate, one finger-like process at dorsal third. Genital style in lateral view (Fig. 21) broad and rounded, dorsal margin concave, dorsal half of posterior margin mound-like, covered with a lot of micro teeth; in posterior view (Fig. 22) short and stout, mostly oval, dorsal angle forked, covered with a lot of micro teeth. Aedeagus in dorsal view (Fig. 23) with basal half broad, apical half abruptly narrowed, stick-like, each side with one spine-like process at widest part; in lateral view (Fig. 24) with base broad, narrowing apically, aedeagal shaft slightly curved ventrad. Connective in lateral view (Fig. 24) curved ventrad, fused with base of aedeagus, nearly forming Y-shape.

#### Type material.

Holotype: ♂, China: Hainan, Diaoluoshan National Natural Reserve (18°47'N, 109°52'E), on bamboo, 9–12 Apr. 2009, X.-H. Hou; paratypes: 1 ♂, 8 ♀♀, data same as holotype.

#### Host plant.

Bamboo.

#### Distribution.

South China (Hainan) (Fig. 37).

#### Remarks.

This new species is similar to the type species from Burma, *Symplanella breviceps* Fennah, 1987, but can be distinguished from the latter in: *i*) frons mostly dark brown (stramineous in *Symplanella breviceps*); *ii*) vertex with anterior margin rounded (angulated in *Symplanella breviceps*); *iii*) posterior margin of pygofer with one spinous process dorsally (absent in *Symplanella breviceps*); *iv*) genital style in posterior view broad and short (narrow and long in *Symplanella breviceps*).

#### Etymology.

The new species is named after the type locality, Hainan Province, China.

### Symplanella zhongtua sp. n.

http://zoobank.org/B8FADA9C-2202-4485-928A-E17811BB1A59

http://species-id.net/wiki/Symplanella_zhongtua

Figs 25–36

#### Measurements.

Body length including forewing: male 6.10–6.35 mm (N = 4), female 6.30–6.50 mm (N = 2); forewing length: male 5.15–5.30 mm (N = 4), female 5.20–5.40 mm (N = 2).

#### Coloration.

General color dirty yellowish brown. Ocelli reddish brown, eyes black brown. Antennae with one black spot at apex of second segment. Frons and clypeus mostly blackish brown. Central area of vertex and pronotum, base of mesonotum with somewhat pale yellowish red. Procoxae, mesocoxae, metapleura, abdominal sternites laterally and pregenital sternite of female fuscous.

#### Head and thorax.

Vertex including eyes narrower than pronotum (0.98:1). Vertex shorter in middle line than broad at base (0.65:1). Frons 1.41 times longer in middle line than widest part. Pronotum as long in middle line as vertex. Mesonotum 1.45 times as long as vertex and pronotum together in middle line. Forewing longer in middle line than broad at widest part (4.45:1).

#### Abdomen.

Anal segment of male in dorsal view (Fig. 30) long oval, widest at apical third, apical margin acutely convex; in lateral view (Fig. 31) with basal half broad, apical half abruptly narrowed, apex slightly swollen, which with some micro teeth at apical ventral margin. Pygofer in lateral view (Fig. 32) with dorsal margin distinctly shorter than ventral margin, posterior margin sinuate, with one stout spine-like process, directed caudo-ventrad. Genital style in lateral view (Fig. 33) with dorsal and ventral angles rounded, posterior margin sinuate; in posterior view (Fig. 34) short and broad, dorsal margin strongly concave, forcipate, inner and outer angles rounded. Aedeagus in ventral view (Fig. 35) with base mostly broad, apical third abruptly narrowed, stick-like, apical margin rounded, each side with one spine-like process; phallobase lobe-like, each with one small spine at apex; aedeagus in lateral view (Fig. 36) with base slightly broad, apical part mostly straight and slender, apical third slightly curved ventrad; phallobase slender, beanpod-like. Connective in lateral view (Fig. 36) narrow and slender, apical half reflexed ventrad and cephalad.

#### Type material.

Holotype: ♂, China: Yunnan, Xishuangbanna, Menglun (21°55'N, 101°13'E), on bamboo, 28 July 2011, W.-B. Zheng and Z.-M. Chang; paratypes: 3 ♂♂, 2 ♀♀, data same as holotype.

#### Host plant.

Bamboo.

#### Distribution.

Southwest China (Yunnan) (Fig. 37).

#### Remarks.

This new species is similar to *Symplanella brevicephala* (Chou, Yuan & Wang, 1994), but can be distinguished by: *i*) frons and clypeus mostly black (dark brown in *Symplanella brevicephala*); *ii*) posterior margin of male pygofer having one stout spinous process at middle, directed ventro-caudad (having one stout tooth-like process dorsally, directed dorso-caudad in *Symplanella brevicephala*); *iii*) genital style in lateral view dorso-apical angle broadly rounded (acutely rounded in *Symplanella brevicephala*); *iv*) aedeagal shaft mostly straight (S-shaped in *Symplanella brevicephala*).

#### Etymology.

The name is derived from transliteration of the Chinese “zhongtu”, meaning posterior margin of male pygofer having one stout spinous process at middle.

## Discussion

**Diversity of bamboo-feeding planthoppers.** The current authors paid particular attention to the species of bamboo planthopper in field research and collected large quantities of specimens in the past twelve years. A number of new taxa or new records were found and some of them have been published (Chen and Yang 2010). Based on the literature and the result of field work, the species diversity of Chinese bamboo-feeding planthoppers is very abundant and more than 84 species (in 18 genera) feed exclusively on Bambusoideae (Che et al. 2009; Chen and Yang 2010; Hou and Chen 2010a, b, c; Chen and Zhang 2011; Yang and Chen 2011; Chang and Chen 2012; Zhang and Chen 2013). They mostly are members of the family Delphacidae (78 species in 15 genera), Caliscelidae (three species in two genera), Cixiidae (two species in one genus) and Tropiduchidae (one species in one genus). The genus *Symplanella* with three known species and three new species described in this paper, represents the second bamboo-feeding genus in the tribe Augilini after *Pseudosymplanella* Che, Zhang & Webb, 2009 (Che et al. 2009).

**Host plant.** As a result of our field research, five species of *Symplanella* from China were found feeding exclusively on bamboo. Unfortunately, no more other information on host plant is available except for *Symplanella recurvata* collected on *Neosinocalamus* sp..

**Distribution.** Based on the literature and the result of field work, five described species within *Symplanella* are distributed in southern China (Chou et al. 1994; Zhang and Wang 2009; this paper) and *Symplanella breviceps* Fennah, 1987 occurring in Burma (Fennah 1987) (Fig. 37). It seems that the members of the genus *Symplanella* are restricted to the Oriental region.

## Acknowledgements

This research was supported by the National Natural Science Foundation of China (No. 30560020, 31060290, 31093430, 31160163) and the International Science and Technology Cooperation Program of Guizhou (No. 20107005).

## Supplementary Material

XML Treatment for Symplanella

XML Treatment for Symplanella recurvata

XML Treatment for Symplanella hainanensis

XML Treatment for Symplanella zhongtua

## References

[B1] Chan M-L Yang C-T (1994) Issidae of Taiwan (Homoptera: Fulgoroidea). Taichung, Taiwan, 188 pp.

[B2] Chang Z-M Chen X-S (2012) *Tambinia bambusana* sp. n., A new bamboo-feeding species of Tambiniini (Hemiptera: Fulgoromorpha: Tropiduchidae) from China. The Florida Entomologist 95: 971-978. doi: 10.1653/024.095.0423

[B3] Che Y-L Zhang Y-L Webb MD (2009) A new genus and species of the planthopper tribe Augilini Baker (Hemiptera, Caliscelidae, Ommatidiotinae) from Thailand and China. Zootaxa 2311: 49-54

[B4] Chen X-S Yang L (2010) Oriental bamboo delphacid planthoppers: three new species of genus *Kakuna* Matsumura (Hemiptera: Fulgoroidea: Delphacidae) from Guizhou Province, China. Zootaxa 2344: 29-38. doi: 10.1603/AN09171

[B5] Chen X-S Zhang Z-G (2011) *Bambusicaliscelis*, a new bamboo-feeding planthopper genus of Caliscelini (Hemiptera: Fulgoroidea: Caliscelidae: Caliscelinae) with descriptions of two new species and their fifth instar nymphs from Southwest China. Annals of the Entomological Society of America 104: 95-104

[B6] Chou I Yuan F Wang YL (1994) A newly recorded genus and three new species of Lophopidae from China (Homoptera: Fulgoroidea). Journal of Northwest Forestry College 9: 44-51

[B7] Fennah RG (1987) A recharacterisation of the Ommatidiotini (Hem.-Hom., Fulgoroidea, Issidae, Caliscelinae) with the description of two new genera. Entomologist’s Monthly Magazine 123: 243-247

[B8] Gnezdilov VM Wilson MR (2006) Systematic notes on tribes in the family Caliscelidae (Hemiptera: Fulgoroidea) with the description of new taxa from Palaearctic and Oriental Regions. Zootaxa 1359: 1-30

[B9] Hou X-H Chen X-S (2010a) Oriental bamboo planthoppers: two new species of the genus *Bambusiphaga* (Hemiptera: Fulgoroidea: Delphacidae) from Hainan Island, China. Florida Entomologist 93: 391-397. doi: 10.1653/024.093.0311

[B10] Hou X-H Chen X-S (2010b) Description of one new species of Oriental bamboo planthopper genus *Arcofacies* Muir (Hemiptera: Fulgoroidea: Delphacidae) from Yunnan, China. Acta Zootaxonomica Sinica 35: 52-56

[B11] Hou X-H Chen X-S (2010c) Review of the Oriental bamboo delphacid genus *Neobelocera* Ding & Yang (Hemiptera: Fulgoroidea: Delphacidae) with the description of one new species. Zootaxa 2387: 39-50

[B12] Yang L Chen X-S (2011) The Oriental bamboo-feeding genus *Bambusiphaga* Huang & Ding, 1979 (Hemiptera: Delphacidae: Tropidocephalini): a checklist, a key to the species and description of two new species. Zootaxa 2879: 50-59

[B13] Zhang P Chen X-S (2013) Two new bamboo-feeding species of the genus *Neocarpia* Tsaur & Hsu (Hemiptera: Fulgoromorpha: Cixiidae: Eucarpiini) from Guizhou Province, China. Zootaxa 3641: 41-48. doi: 10.11646/zootaxa.3641.1.4 10.11646/zootaxa.3641.1.4 26287065

[B14] Zhang L Wang Y (2009) A taxonomic study on the genus *Symplanella* Fennah (Hemiptera: Issidae) from China. Entomotaxonomia 31: 176-180

